# Factors associated with good self-rated health of non-disabled elderly living alone in Japan: a cross-sectional study

**DOI:** 10.1186/1471-2458-7-297

**Published:** 2007-10-22

**Authors:** Wei Sun, Misuzu Watanabe, Yoshimi Tanimoto, Takahiro Shibutani, Rei Kono, Masahisa Saito, Kan Usuda, Koichi Kono

**Affiliations:** 1 Department of Hygiene & Public Health, Osaka Medical College, 2-7 Daigakumachi Takatsuki city, Osaka 569-8686, Japan; 2 School of Public Health, China Medical University, No. 92 Beier road, Heping district, Shenyang 110001, PR China

## Abstract

**Background:**

Self-rated health (SRH) is reported as a reliable predictor of disability and mortality in the aged population and has been studied worldwide to enhance the quality of life of the elderly. Nowadays, the elderly living alone, a particular population at great risk of suffering physical and mental health problems, is increasing rapidly in Japan and could potentially make up the majority of the aged population. However, few data are available pertaining to SRH of this population. Given the fact that sufficient healthcare is provided to the disabled elderly whereas there is little support for non-disabled elderly, we designed this population-based survey to investigate SRH of non-disabled elderly living alone and to identify the factors associated with good SRH with the purpose of aiding health promotion for the elderly.

**Methods:**

A cross-sectional study was conducted in a metropolitan suburb in Japan. Questionnaires pertaining to SRH and physical conditions, lifestyle factors, psychological status, and social activities, were distributed in October 2005 to individuals aged ≥ 65 years and living alone. Response rate was 75.1%. Among these respondents, a total of 600 male and 2587 female respondents were identified as non-disabled elderly living alone and became our subjects. Multivariate logistic regression was used to identify the factors associated with good SRH and sex-specific effect was tested by stepwise logistic regression.

**Results:**

Good SRH was reported by 69.8% of men and 73.8% of women. Multivariate logistic regression analysis showed that good SRH correlated with, in odds ratio sequence, "can go out alone to distant places", no depression, no weight loss, absence of self-rated chronic disease, good chewing ability, and good visual ability in men; whereas with "can go out alone to distant places", absence of self-rated chronic disease, no weight loss, no depression, no risk of falling, independent IADL, good chewing ability, good visual ability, and social integration (attend) in women.

**Conclusion:**

For the non-disabled elderly living alone, sex-appropriate support should be considered by health promotion systems from the view point of SRH. Overall, the ability to go out alone to distant places is crucial to SRH of both men and women.

## Background

Self-rated health (SRH) is a subjective assessment of individual health status and has been well documented as a reliable predictor of functional disability and mortality in aged populations [1-5]. To enhance the quality of life and survival of the elderly, SRH and related determinants have been examined in many populations worldwide. Studies performed in Japan [6-8] showed that SRH worsened with age and correlated with income, physical activity, alcohol consumption, and social support in the community-dwelling 47–77-year-old population and with chewing ability in 80-year-old persons. Foreign surveys [9-13] revealed that chronic disease, functional status, impaired vision, inability to go out alone, physical exercise, health care coverage, and neighborhood also have considerable effects on SRH of the elderly. In addition, sex and rural-urban differences in SRH of the elderly were found in both Japanese and non-Japanese aged population [7,14].

A particular population of elderly individuals, those living alone, is increasing rapidly in Japan. The number exceeded 3 million in 2003, a 39.3% increase over the number in 1998. The Japanese Statistics Bureau reported that, up to 2005, over 15% of the elderly (9.7% of men, 19.0% of women) lived alone. Moreover, ageing of the population and changes of social structure, including longer lifetime, increased mobility of the younger generation, and decreased birth rate, are expected to increase the number of elderly living alone. The elderly living alone could potentially make up the majority of the aged population in Japan. Unfortunately, both cross-sectional [15,16] and longitudinal [17] studies have revealed that the elderly living alone have a greater risk of disability, mental problems and cognitive decline than do those living with a spouse or with others. In light of the great burden this places on society, SRH as a reliable predictor of disability and mortality of the aged should be well studied among the elderly living alone. However, most of SRH studies have focused on the elderly who lives in the community regardless of living arrangement. There are few reports that focus on SRH of the elderly living alone.

The present study was designed to investigate SRH and related factors among the elderly living alone. Given the facts that sufficient healthcare is provided to the disabled elderly and little support is provided to non-disabled elderly and the emphasis The Japanese Ministry of Health, Labor and Welfare has placed on independence of the aged, we initially focused on the non-disabled elderly living alone in Japan. SRH, and the main physical conditions (mobility, visual ability, hearing ability, chewing ability, weight loss, chronic disease, and functional capacity), lifestyle factors (physical exercise and alcohol consumption), psychological status (depression), and social activities (social integration and social networks) were studied in non-disable elderly living alone to assess SRH status and to explore the factors related to good SRH with the purpose to aid the health promotion for aged population living alone.

## Methods

### Definition of "non-disabled elderly living alone"

In Japan, population statistics system categorizes living arrangements only according to the number of persons who live together and have the same family name. Thus, a resident will be registered as "living alone" even if actually living with his/her daughter's family or others with different family name. However, even though an individual actually lives alone, a neighbor may sometimes be his/her sibling or offspring. Therefore, we defined a person "living alone" as one who lives in the community by him/herself and has no any sibling, child, or grandchild living within a radius of 50 meters from his/her house. "Non-disabled elderly living alone" refers to elderly persons who live alone, as we defined, can perform activities of daily living (ADL, i.e. walking, eating, using the toilet, bathing and dressing) independently, have no cognitive impairment, and do not use the long-term care insurance that, since April 2000, has been provided for the elderly who meet particular physical or mental disability criteria.

### Study population

The Welfare Commission is an organization through which the Japanese Ministry of Health, Labour and Welfare provides support for the elderly living alone. Every elderly person living alone is assigned a particular Welfare Commissioner whose responsibility is to offer help whenever a problem arises. According to the data of this organization, there are a total of 5943 residents aged ≥ 65 years that live alone, as we defined, in Takatsuki city, a metropolitan suburb that lies between Osaka city and Kyoto city, Japan. After obtaining the written informed consent about the conduct of the survey, questionnaires were distributed to all these individuals through the Welfare Commissioner in October 2005. We received effective responses from 4465 individuals (response rate 75.1%). Among these respondents, 3187 individuals (600 men and 2587 women) met the criteria for non-disabled elderly living alone, and these individuals became our subjects.

### Assessments of SRH

With referring the study conducted in middle-age Japanese [7], SRH was assessed with a single-item measure: "In general, how do you think about your health?" with 4 possible responses: (1) excellent, (2) good, (3) fair, and (4) poor; and responses were dichotomized as "good" or "poor" by combining response 1 with response 2 and response 3 with response 4.

### Factor selection for the assessment

To our knowledge, this study is the first attempt in Japan to investigate SRH among the non-disable elderly living alone. Physical condition, lifestyle factor, psychological status, and social activity were considered to explore the factors associated with SRH. Although socioeconomic status is known to correlate with SRH, the Law on Personal Information Protection implemented in Japan from April 1, 2005, does not permit us to access information on marital status, education, and income. In Japan, however, as a primary benefit of the social welfare program, all elderly persons are provided a pension regardless of whether they worked when they were young. Thus, income may not be a problem for the elderly. Nevertheless, we still use the question "Do you have to restrict meals because of economic problems?" to estimate the income status of our study population.

With respect to physical condition, the effects on SRH of visual ability, hearing ability, chewing ability, chronic disease, and inability to go out have been studied in previous surveys [8-11]. Therefore, these factors were also assessed in the present study. Given the specific study population, i.e., those living alone, we also included risk of falling to enhance assessment of mobility. Functional status has also been reported to correlate with SRH [9]. Because our study population comprised the non-disabled elderly, we prefer to assess "high-level functional capacity" rather than "basic activities of daily living". Thus, instrumental activities of daily living (IADL) was assessed, as an indicator of "high-level functional capacity" [18]. In addition, because weight loss, especially unintentional weight loss, is common among the elderly and can reflect disease severity or undiagnosed illness, as reviewed by Alibhai SM [19], this was also assessed.

Lifestyle factors such as physical exercise [7,13] and alcohol consumption [7] have been reported to correlate with SRH of the elderly and thereby were examined in this study. Because walking is a particularly feasible form of physical exercise for the elderly, we selected the activity of having a walk to indicate physical exercise. Also, considering that heavy drinking might be rare or not practical for an elderly person, we did not assessed alcohol consumption on the base of the volume of alcohol intake.

Depression is a major psychological disorder that affects the quality of life of the elderly [20,21]. Thus, psychological status was assessed by examining depression in our study population.

As for social activity, social support such as having a confidant with whom to talk about the troubles, a person to take the elderly to the hospital, and a person who can help to prepare meals, has been studied among middle-age Japanese [7]. For our study subjects, the Welfare Commissioner is just the caregiver with such responsibility. Rather than social support, social integration and social networks appear to exert more influence on SRH in this population, therefore, these two items were assessed.

A questionnaire pertaining to SRH and physical condition, lifestyle factor, psychological status, and social activity was distributed to the non-disabled elderly living alone. Two-week reliability of the questionnaire was 90.3%.

### Assessments of physical condition, lifestyle factor, psychological status, and social activity

*Physical conditions *was assessed on the basis of eight items: 1) going out alone to distant places, 2) risk of falling, 3) visual ability, 4) hearing ability, 5) chewing ability, 6) weight loss, 7) self-rated chronic disease, and 8) IADL. The item "going out alone to distant places" was assessed dichotomously as "can" if the individual reported being able to go out to distant places by him/herself or "cannot" if the individual reported being able to go only around the house, being independent indoors but unable to go out without help, or needing some help even indoors, as our previous report [22]. "Risk of falling" was examined on the basis of seven items: tripping or stumbling, inability to cross a road within the time allotted to the green signal, dizziness or faintness, inability to negotiate indoor obstacles, inability to wring out a towel, use of a cane, and knee joint pain. According to Toba K's report [23], it was dichotomized as "no fall risk" when less than three items were positive and "fall risk" when four or more items were positive. For assessment of "visual ability", participants were asked whether they can read the newspaper clearly with or without reading glasses, and for assessment of "hearing ability", participants were asked whether they need voices to be raised for conversation or the volume to be raised for watching television. "Chewing ability" was examined on the basis of the difficulty of eating different foods, as reported by Ikebe [24]. "Weight loss" was examined by asking, "Has your body weight decreased about 5 kg in the past 6 months?", for which we referred the criteria usually used in nutritional assessment in Japan [25]. "Self-rated chronic disease" was assessed dichotomously as "absence" or "presence" if any disease including hypertension, cardiovascular disease, diabetes, or stoke had been diagnosed. "IADL" was determined using the 5-item Tokyo Metropolitan Institute of Gerontology (TMIG) Index of Competence (i.e., using public transportation, shopping for daily necessities, preparing meals, paying bills, and managing deposits) [18]. A full score of five categorized the study subject as independent and a score of 0–4 categorized the subject as dependent [26].

*Lifestyle factors *comprise two items: 1) having a walk regularly, and 2) alcohol consumption. "Having a walk regularly" was assessed according to the frequency of taking walks as "≥ 1~2 times/week" or "rarely". Alcohol consumption was determined with the questions "Do you drink heavily?" and "Do you have 3 or more drinks of beer, sake or wine almost every day?".

*Psychological status *referred to depression. A simple Geriatric Depression Scale (GDS – 5 items) [27] was used to assess depression among the participants. Each item was coded as 0 if negative or 1 if positive. Score ≥ 2 was used to diagnose the presence of depressive symptoms, as reported by Rinaldi P [28].

*Social activities *comprised two items: 1) social integration, and 2) social networks. "Social integration" was assessed according to whether respondents participated in (a) a welfare center association for the aged, (b) recreational activities at a public hall or community center, or (c) a hobby association. "Social integration" was described as "attend" if the respondent reported participating in (a) or (b) at least weekly or in (c) at least monthly and as "does not attend" if not. "Social networks" was assessed according to the frequency of visual or nonvisual contact (such as by telephone, fax or mail) with relatives, friends, or confidants as ≥ 1–2 times/week [29].

### Statistical analysis

Among all independent variables, items to which over 95% of individuals have the same responses were not included in the data analysis. Income estimation (no economic problem) and alcohol consumption (no heavy drinking) were thereby excluded.

The distribution of SRH in relation to age, sex, and various physical, lifestyle, psychological, and social items was examined by chi-square test. The factors associated with good SRH were identified by multivariate logistic regression with adjustment for age. The multivariate logistic regression analysis was performed in two steps: 1) fit univariate model with each variable. All variables were entered in the model separately with adjustment for age, and any variable that was significant at the 0.25 level was a potential candidate for the multivariate model. 2) Fit multivariate model. All items selected from 1) step were entered in the model. Items with *p *> 0.05 were eliminated one at a time in the sequence of *p *value. When an item was eliminated, if the change of any remaining parameter estimate was greater than 20%, this item would remain in the model as a confounder. In this study, all independent variables were found to be significant in 1) step and no confounders were found during item elimination.

Sex-specific associations with SRH were examined by stepwise multivariate logistic regression. The model included all risk factors identified as described above and the interaction terms of sex with each risk factor.

In view of the prominent difference in life expectancy between men and women (78.6 years for men; 85.5 years for women) and the sex-specific results, we performed statistical analyses not only for the total study population but also separately for men and women.

All data was shown as number (N), prevalence (%), odds ratio (OR), and 95% confidence interval (95%CI). SAS for Windows, Ver. 8.2, was used for all statistical analyses.

## Results

Overall, 73.1% of individuals reported a good SRH. The distribution of SRH by sex and by age is shown in Table 1. 69.8% of men and 73.8% of women reported a good SRH (*p *< 0.05). Among women, good SRH responses were significantly more frequent in the 65–74 years age group than in the 75+ years age group (*p *< 0.05), but this difference was not found in men. In the 65–74 years age group, good SRH responses were significantly more frequent in women than in men (*p *< 0.05), whereas there was no sex difference in the 75+ years age group.

**Table 1:** The distribution of SRH by sex and by age among non-disabled elderly living alone.

	Men (N = 600)		Women (N = 2587)		*p*
		
	Good	Poor	Good	Poor	
	N (%)	N (%)	N (%)	N (%)	
Age					
65~74	207(69.5)	91(30.5)	991(75.6)	319(24.4)	<0.05
75+	212(70.2)	90(29.8)	919(72.0)	358(28.0)	ns
*p*		ns		<0.05	

Total	419(69.8)	181(30.2)	1910(73.8)	677(26.2)	<0.05

ns: not significant.

Results of chi-square analysis of good SRH in relation to various physical, lifestyle, psychological, and social factors are shown in Table 2. Among the total study population and women, all physical, lifestyle, psychological, and social items were associated with good SRH. Among men, however, all items except IADL and social integration were related to good SRH. Of all factors analyzed, "going out alone to distant places" had the highest OR in total study population (OR = 5.02), men (OR = 6.09) and women (OR = 5.11).

**Table 2:** Univariate analysis of the SRH in relation to various physical, lifestyle, psychological and social factors.

Variables	Total		Men		Women	
	
	Good SRH N(%)	OR (95%CI)	Good SRH N(%)	OR (95%CI)	Good SRH N(%)	OR (95%CI)
**Physical conditions:**						
Going out alone to distant places						
Cannot	281(44.6)		26(33.3)		255(46.2)	
Can	2047(80.2)	5.02(4.18–6.04)	393(75.3)	6.09(3.66–10.16)	1654(81.4)	5.11(4.18–6.25)
Risk of falling						
Fall risk	1822(70.3)		312(66.7)		1510(71.1)	
No fall risk	506(85.5)	2.48(1.95–3.17)	107(81.7)	2.23(1.38–3.61)	399(86.6)	2.61(1.97–3.47)
Visual ability						
Impaired	193(48.3)		37(43.5)		156(49.5)	
Good	2132(76.7)	3.52(2.84–4.37)	382(74.2)	3.73(2.32–5.97)	1750(77.2)	3.46(2.71–4.40)
Hearing ability						
Impaired	114(51.4)		27(48.2)		87(52.4)	
Good	2202(74.8)	2.81(2.13–3.71)	391(72.3)	2.80(1.60–4.89)	1811(75.4)	2.78(2.02–3.82)
Chewing ability						
Limited	147(50.3)		40(49.4)		107(50.7)	
Good	2182(75.4)	3.02(2.36–3.85)	379(73.0)	2.77(1.72–4.47)	1803(75.9)	3.06(2.30–4.07)
Weight loss≅5 kg in past 6 months						
Loss	94(43.7)		21(42.9)		73(44.0)	
No loss	2206(75.6)	3.98(3.00–5.28)	392(72.7)	3.56(1.96–6.46)	1814(76.2)	4.08(2.96–5.63)
Self-rated chronic disease						
Presence	1097(64.2)		224(62.0)		873(64.7)	
Absence	1232(83.4)	2.81(2.37–3.33)	195(81.6)	2.71(1.84–4.00)	1037(83.8)	2.81(2.33–3.39)
IADL (TMIG-index)						
Dependent	91(43.5)		24(57.1)		67(40.1)	
Independent	2238(75.2)	3.93(2.95–5.23)	395(70.8)		1843(76.2)	4.78(3.46–6.61)
**Lifestyle factor:**						
Having a walk regularly						
Rarely	603(67.3)		109(63.7)		494(68.1)	
≥1~2 times/week	1682(75.6)	1.51(1.27–1.79)	304(72.4)	1.49(1.02–2.18)	1378(76.4)	1.51(1.25–1.83)
**Psychological status:**						
GDS-5 score						
Depression	559(55.5)		124(52.8)		435(56.3)	
No depression	1726(82.1)	3.68(3.11–4.35)	282(82.2)	4.14(2.84–6.03)	1444(82.1)	3.55(2.94–4.28)
**Social activities:**						
Social integration						
Does not attend	1405(68.6)		312(68.4)		1093(68.7)	
Attend	901(81.6)	2.03(1.70–2.42)	105(75.0)		796(82.6)	2.16(1.77–2.63)
Social networks						
Rare contact	253(62.2)		111(63.4)		142(61.2)	
Contact ≥ 1~2 times/week	2067(75.0)	1.82(1.47–2.27)	306(72.9)	1.55(1.06–2.25)	1761(75.4)	1.94(1.46–2.56)

Results of multivariate logistic regression analyses in the total study population are shown in Table 3. Age was fixed in the model as a continuous variable. The items associated with SRH were, in OR sequence, "going out to distant places", self-rated chronic disease, GDS, weight loss, chewing ability, risk of falling, visual ability, hearing ability, and social integration.

**Table 3:** Multivariate logistic regression analysis of the main factors associated with good SRH. (N = 3025)

Variables	OR	
	
	Value	95%CI
Age (fixed)		
Going out alone to distant places (can/cannot)	3.72	2.97–4.65
Self-rated chronic disease (absence/presence)	2.61	2.15–3.18
GDS-5 score (no depression/depression)	2.45	2.02–2.96
Weight loss ≅5 kg in past 6 months (no loss/loss)	2.41	1.74–3.34
Chewing ability (good/limited)	1.89	1.40–2.54
Risk of falling (no fall risk/fall risk)	1.81	1.37–2.40
Visual ability (good/impaired)	1.71	1.31–2.24
Hearing ability (good/impaired)	1.50	1.06–2.13
Social integration (attend/does not attend)	1.40	1.14–1.72

-2 Log L: 2799.097 Hosmer and Lemeshow Goodness-of-Fit Test: *p *= 0.85

Age is treated as continuous variable

Results of stepwise multivariate logistic regression analysis are shown in Table 4. Sex-specific associations with good SRH were examined by entering all risk factors and interaction terms of sex with each risk factor in the model with adjustment for age. Of all risk factors, self-rated chronic disease and social integration were found to have stronger associations with SRH in women than in men.

**Table 4:** Stepwise multivariate logistic regression analysis of sex-specific associations with good SRH.

Variables	OR	
	
	Value	95%CI
Age (fixed)		
Going out alone to distant places (can/cannot)	3.81	3.04–4.77
GDS-5 score (no depression/depression)	2.43	2.01–2.94
Weight loss ≅5 kg in past 6 months (no loss/loss)	2.38	1.72–3.29
Chewing ability (good/limited)	1.84	1.37–2.47
Risk of falling (no fall risk/fall risk)	1.83	1.38–2.42
Visual ability (good/impaired)	1.71	1.31–2.24
Sex (women/men)*Self-rated Chronic disease (absence/presence)	1.66	1.49–1.84
Hearing ability (good/impaired)	1.47	1.04–2.09
Sex (women/men)*Social integration (attend/does not attend)	1.19	1.06–1.32

-2 Log L: 2803.98 Hosmer and Lemeshow Goodness-of-Fit Test: *p *= 0.73

Age is treated as continuous variable

Results of multivariate logistic regression analyses in men and in women are shown in Tables 5 and 6. With adjustment for age, good SRH was associated with, in OR sequence, "can go out alone to distant places", no depression, no weight loss, absence of self-rated chronic disease, good chewing ability, and good visual ability in men; and with "can go out alone to distant places", absence of self-rated chronic disease, no weight loss, no depression, no risk of falling, independent IADL, good chewing ability, good visual ability, and social integration (attend) in women.

**Table 5:** Multivariate logistic regression analysis of the main factors associated with good SRH in men. (N = 572)

Variables	OR	
	
	Value	95%CI
Age (fixed)		
Going out alone to distant places (can/cannot)	4.12	2.23–7.62
GDS-5 score (no depression/depression)	3.00	1.98–4.55
Weight loss ≅5 kg in past 6 months (no loss/loss)	2.68	1.35–5.31
Self-rated chronic disease (absence/presence)	2.31	1.49–3.59
Chewing ability (good/limited)	2.16	1.23–3.79
Visual ability (good/impaired)	1.91	1.08–3.36

-2 Log L: 563.89 Hosmer and Lemeshow Goodness-of-Fit Test: *p *= 0.73

Age is treated as continuous variable

**Table 6:** Multivariate logistic regression analysis of the main factors associated with good SRH in women. (N = 2471)

Variables	OR	
	
	Value	95%CI
Age (fixed)		
Going out alone to distant places (can/cannot)	3.33	2.57–4.31
Self-rated chronic disease (absence/presence)	2.73	2.19–3.40
Weight loss ≅5 kg in past 6 months (no loss/loss)	2.38	1.65–3.43
GDS-5 score (no depression/depression)	2.29	1.85–2.85
Risk of falling (no fall risk/fall risk)	2.01	1.45–2.78
IADL (independent/dependent)	1.89	1.23–2.89
Chewing ability (good/limited)	1.81	1.28–2.56
Visual ability (good/impaired)	1.73	1.29–2.33
Social integration (attend/does not attend)	1.44	1.15–1.81

-2 Log L: 2247.57 Hosmer and Lemeshow Goodness-of-Fit Test: *p *= 0.71

Age is treated as continuous variable

## Discussion

Of the non-disabled elderly living alone, women (73.8%) tended to report good SRH more than men (69.8%). The percentages of good SRH responses in both men and women were considerably higher than that (53%) obtained from community-dwelling Japanese aged 47–77 years [7]. Also, the age-related decrease in SRH, especially in men, was not as obvious as that reported by Liang [6]. These discrepant results could be due to the fact that none of our subjects were functionally disabled even though they were elderly.

Physical condition, especially the presence of chronic disease, has consistently been documented as the main determinant of SRH of the elderly [9-11]. Our study also revealed correlation between SRH and various physical condition variables, such as the ability to go out alone to distant places, presence or absence of chronic disease, presence or absence of weight loss, visual ability, and chewing ability. Interestingly, for our study population, both men and women, the ability to go out alone to distant places, rather than the present or absence of chronic disease, correlated most strongly with SRH, although chronic disease was also important. For the non-disabled elderly living alone, this ability seems to be a determinant of independent living. Those who maintain an ability to go out alone to distant places are able to perform daily and social activities independently and keep from using long-term care insurance. Therefore, the ability to go out alone to distant places could represent the health status of individuals in this population. The ability to go out alone to distant places was the original criterion proposed by the Ministry of Health, Labour and Welfare in Japan to assess independence in daily life of the disabled elderly. Recently, with the focus on health promotion strategies in Japan, this ability has been used as an indicator of mobility of the elderly, regardless of whether they are disabled. An elderly person who cannot go out alone to distant places cannot walk 1 kilometer continuously [22], has no confidence toward life and seldom participates in social activities [30], and tends to be frail [31]. In our opinion, the ability to go out alone to distant places could also be a good indictor of the health status of the non-disabled elderly living alone. Elderly persons living alone but without the ability to go out alone to distant places might tend to use long-term care insurance and become dependent.

As for psychological health, depression is a common psychological disorder among the elderly and has a great adverse effect on mobility, functional ability, and well-being [20,21]. The prevalence of depression in later life has been reported to range from 0.9% to 42% [32]. Among our subjects, the prevalence of depression was 40.7% in men and 30.5% in women, both in the upper reported range even though none of the participants were disabled and a Welfare Commissioner was available. In view of one study [33] that confirmed an increased prevalence of depression among elderly Japanese living alone than that among those living with a spouse or with others, these findings suggest that depression occurs easily in non-disabled elderly living alone. Correspondingly, a considerable impact of depression on SRH was found in the present study even after controlling for various confounding factors. Although they can still live independently, the elderly living alone seem to sink into loneliness more easily than other elderly persons do. This emotion is able to reduce satisfaction with their health status, which in turn influences physical function and, as a result, impairs their quality of life. Therefore, how to lessen the depression of the elderly living alone is an important question.

Social activity is another interesting topic in the field of geriatrics because of its effect on health and mortality [34,35]. Some studies have linked social integration or social networks with SRH [7,13,36]. We also found correlation between social activities and SRH among non-disabled elderly living alone. However, in the multivariate model that included physical and psychological factors, the effect of social activities was significant only in women, not in men, and pertained mainly to social integration. This sex difference found in our study coincides with the results obtained in a Japanese community-dwelling population [7]. The reason is unclear. The difference might be due to traditional roles assumed by Japanese: the majority of women are limited to housework, whereas most men work away from home. Thus, social contact differs between women and men. Women are more likely to rely on fellowship, whereas men are more likely to rely on formal task/activity relationships. As a result, participation in various voluntary associations is more common among Japanese women. As shown in our study, involvement in social activities is close to 15% higher in women (37.7%) than in men (23.5%). Therefore, such involvement could be more helpful in keeping women, rather than men, from social isolation, and its contribution to SRH of women was observed.

There are several limitations to the present study. First, socioeconomic status was not assessable because of restrictions imposed by the Law on Personal Information Protection, and may have affected the results. Second, chronic disease was self-reported by the subjects and we focused it on lifestyle-related illness, which might affect the accuracy of information and weaken the effect of chronic disease on SRH. Third, alcohol consumption was not assessed quantitatively, which may have lead to the over 95% response to "no heavy drinking". Fourth, our study was a cross-sectional design. It is difficult to draw conclusions about any causal relations between SRH and the factors identified in our study. All findings need to be confirmed in a longitudinal study.

## Conclusion

The present study is the first to assess SRH and related factors among the elderly living alone in Japan. Our findings show that the factors associated with good SRH of non-disabled elderly living alone differ between men and women. Physical condition and psychological status seem to have a considerable effect on SRH of both men and women, whereas social activity seems to have an effect only in women. Overall, the ability to go out alone to distant places is a crucial factor affecting SRH of the non-disabled elderly, both men and women, who live alone.

## Competing interests

The author(s) declare that they have no competing interests.

## Authors' contributions

MW designed the study and conducted the survey. WS participated to the conduct of the survey, performed all statistical analyses and wrote the manuscript. MW and KK supervised the data analysis and the manuscript preparation. YT, TS, RK, MS contributed to the conduct of the study. KU contributed to the interpretation of the data. All authors have read and approved the final manuscript.

## Pre-publication history

The pre-publication history for this paper can be accessed here:


